# Supplementary material for: Investigating online psychological treatment for adolescents with a visible difference in the Dutch YP Face IT study: protocol of a randomised controlled trial
Source: BMJ Open. 2021 Jan 22;11(1):e041449. doi: 10.1136/bmjopen-2020-041449 (PMC7831738; doi:10.1136/bmjopen-2020-041449)
Supplement: Author's manuscript [file bmjopen-2020-041449.draft_revisions.pdf]

BMJ Open

BMJ Open is committed to open peer review. As part of this commitment we make the peer review history of every article we publish publicly available.

When an article is published we post the peer reviewers' comments and the authors' responses online. We also post the versions of the paper that were used during peer review. These are the versions that the peer review comments apply to.

The versions of the paper that follow are the versions that were submitted during the peer review process. They are not the versions of record or the final published versions. They should not be cited or distributed as the published version of this manuscript.

BMJ Open is an open access journal and the full, final, typeset and author-corrected version of record of the manuscript is available on our site with no access controls, subscription charges or pay-per-view fees (http://bmjopen.bmj.com).

If you have any questions on BMJ Open's open peer review process please email info.bmjopen@bmj.com

BMJ Open

The Dutch YP Face IT Study, investigating online psychological treatment for Adolescents with a Visible Difference: Study Protocol of a Randomised Controlled Trial

Journal:	*BMJ Open*
Manuscript ID	bmjopen-2020-041449
Article Type:	Protocol
Date Submitted by the Author:	09-Jun-2020

Complete List of Authors:	van Dalen, Marije; Erasmus MC Sophia Children Hospital, Department of Child and Adolescent Psychiatry/Psychology<br>Pasmans, Suzanne G. M. A.; Erasmus MC Sophia Children Hospital, Department of Dermatology<br>Aendekerk, Marie-Louise; Erasmus MC Sophia Children Hospital, Department of Child and Adolescent Psychiatry/Psychology<br>Mathijssen, Irene; Erasmus MC Sophia Children Hospital, Department of Plastic and Reconstructive Surgery<br>Koudstaal, Maarten; Erasmus MC Sophia Children Hospital, Department of Oral and Maxillofacial Surgery<br>Timman, Reinier; Erasmus MC, Department of Psychiatry, Section of Medical Psychology & Psychotherapy<br>Williamson, Heidi; University of the West of England, Health and Life Sciences<br>Hillegers, Manon; Erasmus MC Sophia Children Hospital, Department of Child and Adolescent Psychiatry/Psychology<br>Utens, Elisabeth; Erasmus MC Sophia Children Hospital, Department of Child and Adolescent Psychiatry/Psychology<br>Okkerse, Jolanda; Erasmus MC Sophia Children Hospital, Department of Child and Adolescent Psychiatry/Psychology

Keywords:	Child & adolescent psychiatry < PSYCHIATRY, Anxiety disorders < PSYCHIATRY, Depression & mood disorders < PSYCHIATRY

SCHOLARONE™
Manuscripts

I, the Submitting Author has the right to grant and does grant on behalf of all authors of the Work (as defined in the below author licence), an exclusive licence and/or a non-exclusive licence for contributions from authors who are: i) UK Crown employees; ii) where BMJ has agreed a CC-BY licence shall apply, and/or iii) in accordance with the terms applicable for US Federal Government officers or employees acting as part of their official duties; on a worldwide, perpetual, irrevocable, royalty-free basis to BMJ Publishing Group Ltd ("BMJ") its licensees and where the relevant Journal is co-owned by BMJ to the co-owners of the Journal, to publish the Work in this journal and any other BMJ products and to exploit all rights, as set out in our licence.

The Submitting Author accepts and understands that any supply made under these terms is made by BMJ to the Submitting Author unless you are acting as an employee on behalf of your employer or a postgraduate student of an affiliated institution which is paying any applicable article publishing charge ("APC") for Open Access articles. Where the Submitting Author wishes to make the Work available on an Open Access basis (and intends to pay the relevant APC), the terms of reuse of such Open Access shall be governed by a Creative Commons licence – details of these licences and which Creative Commons licence will apply to this Work are set out in our licence referred to above.

Other than as permitted in any relevant BMJ Author's Self Archiving Policies, I confirm this Work has not been accepted for publication elsewhere, is not being considered for publication elsewhere and does not duplicate material already published. I confirm all authors consent to publication of this Work and authorise the granting of this licence.

For peer review only

Running head: Dutch YP FACE IT STUDY PROTOCOL

The Dutch YP Face IT Study, investigating online psychological treatment for Adolescents with a Visible Difference: Study Protocol of a Randomised Controlled Trial

M. van Dalen[1], S.G.M.A. Pasmans[2], E.W.C. Aendekerk[1], I.M.J. Mathijssen[3], M.J. Koudstaal[4], R. Timman[5], H. Williamson[6], M.H.J. Hillegers[1], E.M.W.J. Utens[1,7,8], J.M.E. Okkerse[1]

1. Department of Child and Adolescent Psychiatry/Psychology, Erasmus MC-Sophia Children's Hospital, Rotterdam, The Netherlands. 2. Department of Dermatology, Centre of Pediatric Dermatology, Erasmus MC-Sophia Children's Hospital, Rotterdam, The Netherlands. 3. Department of Plastic and Reconstructive Surgery and Hand Surgery, The Dutch Craniofacial Centre, Erasmus MC-Sophia Children's Hospital, Rotterdam, The Netherlands. 4. Department of Oral and Maxillofacial Surgery, The Dutch Craniofacial Centre, Erasmus MC-Sophia Children's Hospital, Rotterdam, The Netherlands. 5. Department of Psychiatry, unit of Medical Psychology and Psychotherapy, Erasmus MC, Rotterdam, The Netherlands. 6. Department of Health and Social Sciences, University of the West of England, Bristol, United Kingdom 7.Research Institute of Child Development and Education, University of Amsterdam, Amsterdam, The Netherlands. 8. Academic Center for Child Psychiatry the Bascule/ Department of Child and Adolescent Psychiatry, Amsterdam University Medical Center, Amsterdam, The Netherlands.

Corresponding author:

Dr. Jolanda Okkerse

Department of Child and Adolescent Psychiatry/Psychology

Erasmus MC-Sophia Children's Hospital, P.O. Box 2060,

3000 CB Rotterdam, The Netherlands

E-mail: j.okkerse@erasmusmc.nl

Tel.: 0031 (0) 10 704 02 09

Word count (excluding title page, abstract, references, figures and tables, max. 4000): 3759

Author e-mail addresses:

Marije van Dalen	m.vandalen.1@erasmusmc.nl
Suzanne G.M.A. Pasmans	s.pasmans@erasmusmc.nl
E.W.C. (Marie-Louise) Aendekerk	e.aendekerk@erasmusmc.nl
Irene M.J. Mathijssen	i.mathijssen@erasmusmc.nl
Maarten J. Koudstaal	m.koudstaal@erasmusmc.nl
Reinier Timman	r.timman@erasmusmc.nl
Heidi Williamson	heidi3.williamson@uwe.ac.uk
Manon H.J. Hillegers	m.hillegers@erasmusmc.nl
Elisabeth M.W.J. Utens	e.utens@erasmusmc.nl
Jolanda M.E. Okkerse	j.okkerse@erasmusmc.nl

For peer review only

Abstract

Introduction: This paper outlines the study protocol for the Dutch YP Face IT study. Adolescents with a visible difference (i.e. disfigurement) often experience challenging social situations such as being stared at, receiving unwanted questions or being teased. As a consequence, some of these adolescents suffer adverse psychosocial outcomes and appearance-related distress. To address this appearance-related distress, an online psychotherapeutic intervention, YP Face IT, has been developed. YP Face IT uses Social Skills Interaction Training and Cognitive Behavioural Therapy. The Dutch YP Face IT study tests whether this intervention is effective in reducing social anxiety and improving body esteem. **Methods and analysis:** Participants are adolescents aged 12-18 with a visible difference and suffering from appearance-related distress. A total of 224 adolescents will be randomised to either the care as usual or the YP Face IT condition. Adolescents will complete questionnaires at baseline, at 13 weeks and at 25 weeks. **Ethics and dissemination:** Research ethics approval was obtained from the Medical Ethics review Committee Rotterdam (reference number MEC-2018-052/NL63955.078.18). Findings will be disseminated through academic peer-reviewed publications, conferences and in newsletters to patient associations and participants of the study. **Trial registration:** This trial was registered in The Netherlands trial register, NL7626, Registered 26th March 2019, https://www.trialregister.nl/trial/7626.

Keywords: Visible difference; appearance-related distress; adolescents; anxiety; body-esteem

For peer review only

ARTICLE SUMMARY

Strengths and limitations of this study

- This is the first Randomised Controlled Trial (RCT) to assess YP Face IT
- Participants are adolescents with a visible difference, aged 12 to 18 years, throughout The Netherlands
- Participants are randomised to Care As Usual (CAU) or YP Face IT, after assessment of their scores on self-report measures of mental health at baseline
- Standardized quantitative self-report measures will be used to collect data at three time-points, including a 3-month follow-up
- Participants are not blinded to allocation, which could lead to reporting bias

INTRODUCTION

Having a visible difference can have a substantial impact on one's life. People with a visible difference commonly encounter difficult social situations, such as being stared at, receiving unwanted questions and feeling isolated. A visible difference (i.e. disfigurement) to the face or body occurs in 1 in 44 people, a visible difference solely to the facial area in 1 in 111(1). A visible difference can be either congenital (e.g. cleft lip and palate, craniofacial conditions), result from a skin condition (e.g. vitiligo, psoriasis and acne), trauma (e.g. burns, scars), disease (e.g. cancer, meningitis and alopecia areata) or medical treatment (e.g. surgery or radiotherapy). Whether someone identifies as having a visible difference is a subjective experience. A clear definition of what a visible difference is can thus not be given.

Some studies show that adolescents with a visible difference experience no additional psychosocial problems(2, 3) and even perceive their friendship, social acceptance, appearance and emotional distress in a more positive way than regular peers(4). However, reviews show

For peer review only

that a significant amount of adolescents with a visible difference do experience adverse psychosocial outcomes(5-7). More specifically, they experience higher levels of minor psychological disturbance, self-consciousness in public situations, neuroticism(8), internalising symptoms(7, 9) and anxiety, depression and difficulties with social functioning(10). Furthermore, parents perceive their children as being more shy and inhibited(11).

Available interventions for adolescents or adults with a visible difference are scarce and lack evidence-base(12-15). Research often lacks a solid design and overall methodological quality, and yields small effect sizes. However, there is some support for models involving Social Skills Training (SST), Cognitive Behavioural Therapy (CBT; 12, 14, 15) and self-help for managing anxiety(13).

YP (Young People) Face IT was developed in the UK, in collaboration with adolescents and the charity Changing Faces(16). YP Face IT was derived from the adult intervention Face IT which was found to be effective at reducing anxiety, depression and appearance concerns whilst increasing positive adjustment(17). In an acceptability and feasibility study concerning YP Face IT, the intervention was found to be acceptable, with most of the adolescents thinking it would help them improve their confidence and self-acceptance and help them develop new skills in dealing with difficult social situations(16).

Aims

In sum, interventions aimed at adolescents with a visible difference are scarce and have limited evidence to support them. The available evidence, however, does point to SST and CBT as being potentially effective methods for managing psychosocial difficulties amongst people with a visible difference. In an attempt to provide adolescents coping with a visible difference with an effective intervention focussing on improving emotional resilience,

For peer review only

the Dutch YP Face IT study aims to test an online psychotherapeutic intervention combining Social Interaction Skills Training (SIST) and CBT. More specifically, the aims of the current study is to test the effectiveness of the Dutch version of YP Face IT in reducing anxiety and improving body-esteem amongst Dutch adolescents with a visible difference.

METHODS AND ANALYSIS

To assess the effectivity of the Dutch version of YP Face IT, a randomised controlled trial (RCT) will be conducted. This trial was registered prospectively in the Netherlands Trial Register (NL7626).

Participants

This study will be conducted in a Dutch academic hospital. The target group for this study is Dutch adolescents aged 12-18 with a visible difference and suffering from appearance-related distress. Recruitment will take place throughout The Netherlands.

Adolescents will be eligible for participation if they are aged between 12 and 18, have a visible difference, access to a computer with internet and experience appearance-related distress. The appearance-related distress is operationalised as an elevated score on either social anxiety or depression, or a lower score on body esteem.

Exclusion criteria are a mental disability, reading ability below 12 years of age, visual impairments preventing comprehension of the online intervention, a clinical diagnosis of depression, psychosis, body dysmorphic disorder or an eating disorder, and insufficient proficiency of Dutch. Furthermore, adolescents who receive face-to-face care by a psychologist will be excluded from participation.

Intervention

For peer review only

YP Face IT (Dutch: *Face IT voor jongeren*) is an online psychotherapeutic intervention(16) specifically designed for adolescents and developed in cooperation with adolescents and the British charity Changing Faces. The intervention is aimed at helping adolescents cope with some key concerns people with a visible difference may have, such as anxiety, depression, low self-esteem and appearance-related distress.

YP Face IT combines SIST with CBT. The intervention consists of seven weekly sessions and an additional booster session after six weeks, helping the adolescent recall what was learned during the weekly sessions. The first three sessions focus on SIST, sessions 4-6 focus on CBT and session 7 is a summary of all learned techniques. Each session has different exercises and activities and takes 45-60 minutes to complete. In addition to the weekly sessions, participants will also be asked to complete small homework assignments between sessions. Participants will have access to an online journal in which they can write about their experiences and enter the answers to the homework assignments. Participants will have the opportunity to contact the research team if they are struggling with the homework activities. A reminder e-mail is send if the participant has not entered data relating to the homework activity in their journal within five days following their last session.

Monitoring will take place to assess whether adolescents complete the homework assessments and whether they are struggling in any way with the intervention or their mental health. Monitoring will be done by an experienced clinical psychologist and a master's student. Should we find that adolescents experience severe complaints, they are referred to other care that may better suit their needs.

Care As Usual (CAU).

CAU will consist of regular medical care. The type of the care may differ depending on the condition (e.g. skin care for eczema, orthodontics for cleft palate). Psychological care

For peer review only

is not part of CAU. However, medical personnel will monitor the adolescents' well-being as part of CAU. They may refer the adolescents to a psychologist in case of suspicions of psychological problems. Adolescents that receive psychological care during participation in the study will not be allowed further participation.

Materials

All outcome measures are completed at all three time points.

Primary outcomes

Social anxiety. The Social Anxiety Scale for Adolescents (SAS-A; 18) will be used to assess social anxiety. The SAS-A contains 22 statements that are rated on a 5-point Likert scale (e.g. I worry about what others say about me; I get nervous when I meet new people). A high score indicates high levels of social anxiety. The SAS-A contains three subscales, namely Fear of Negative Evaluation (FNE), social avoidance and distress in new situations (SAD – new), and social avoidance and distress in general (SAD-general). Cronbach's alphas are satisfactory for the subscales, being $\alpha = 0.91$, 0.83 and 0.76 respectively (18). Further research also yielded satisfactory Cronbach's alpha's of $\alpha = 0.89$, 0.80 and 0.70 for FNE, SAD-new and SAD-general respectively (19).

Body esteem. The subscale 'appearance' from the Body Esteem Scale For Adolescents and Adults (BESAA; 20) is used to assess participants attitudes and feelings about their appearance. A higher score indicates more satisfaction with one's appearance. This subscale consists of 10 statements, such as "I like how I look in photos". The items are rated on a 5-point Likert scale ranging from "never" to "always". The subscale demonstrates excellent internal consistency in a sample of American girls ($\alpha = 0.92$) and boys ($\alpha = 0.90$; 21) and in a sample of Dutch university students ($\alpha = 0.90$; 22).

For peer review only

177 Secondary outcomes

178 *Aspects of self-worth.* The Dutch version of the Self-Perception Profiles for
179 Adolescents (SPPA; 23), the Competentiebelevingsschaal voor Adolescenten (CBSA; 24),
180 will be used to assess different aspects of self-worth. The CBSA consists of eight specific
181 domains and a global self-worth subscale. The specific domains are: scholastic competence,
182 social competence, athletic competence, physical appearance, job competence, romantic
183 appeal, behavioral conduct and close friendship. The CBSA consists of 35 items, each
184 consisting of two contrasting statements (e.g. some teenagers are *not* happy with the way they
185 look BUT other teenagers *are* happy with the way they look). The adolescent is asked to pick
186 the statement that is most representative of them and choose whether the statement is 'sort of
187 true for me' or 'really true for me'. All domains demonstrate good to excellent internal
188 reliabilities, ranging from $\alpha = 0.74$ to $\alpha = 0.93$. The global self-worth subscale also
189 demonstrates excellent reliability, with $\alpha = 0.80$-0.89(23).

190 *Perceived stigmatisation.* The Perceived Stigmatization Questionnaire (PSQ; 25) will
191 be used to measure stigmatisation behaviours commonly experienced by people with a visible
192 difference. The PSQ is divided into three subscales, namely: absence of friendly behaviour,
193 confused/staring behaviour and hostile behaviour. These subscales amount to a total of 21
194 items concerning stigmatising behaviours, such as "people avoid looking at me" and "people
195 call me names". Participants are asked to rate how often they experience certain behaviour on
196 a 5-point Likert scale. A high score reflects high perceived stigmatisation. The three subscales
197 have been established(26) and the PSQ has adequate reliability in a sample of children and
198 adolescents with a visible difference ($\alpha = 0.81$; 27).

199 *Health-related quality of life.* The EuroQol-5D-5L (EQ-5D-5L; 28) is used to assess
200 health-related quality of life. The scale has five dimensions, namely: Mobility, Self-Care,

201 Usual Activities, Pain/Discomfort and Anxiety/Depression. Each dimension can be assessed
202 on five levels, which range from no problems to extreme problems. The EQ-5D-5L has good
203 absolute and relative discriminatory power and good convergent validity(29).

204 *Life Engagement.* The Life Engagement Scale(30) will be used to measure the extent
205 that worries or feeling bad about the way you look stop you from engaging in life activities
206 such as going to a social event or doing sports. The scale consists of ten questions on which
207 the adolescent can rate how much their worries or feeling bad about the way they look
208 stopped them from doing activities on a 4-point scale. A high score indicates less life
209 engagement. This instrument was purposively constructed for adolescents with a visible
210 difference. The scale has good internal consistency, with $\alpha = 0.93$ for girls and $\alpha = 0.96$ for
211 boys(30).

212 *Appearance-related distress.* The Mirror, Mirror.. (Spiegeltje, spiegeltje..; 31)
213 questionnaire will be used to assess appearance-related distress. This questionnaire consists of
214 58 statements about appearance (e.g. my appearance makes me insecure), with a 5-point
215 Likert scale ranging from “never” to “always”. The questionnaire also contains a section with
216 38 body-parts where adolescents have to indicate on a 5-point Likert scale how happy they are
217 with said body part (e.g. skin colour, mouth). A high score indicates more dissatisfaction with
218 appearance. As this questionnaire is new and unpublished, it has not been validated. We aim
219 to validate this questionnaire using the data obtained in this study.

220 *Depressive symptoms.* The Child Depression Inventory – 2 (CDI-2; 32) will be used to
221 assess depressive symptoms. The questionnaire consists of 28 items with three answer options
222 (e.g., I am sad sometimes, I am often sad, I am always sad). The scale consists of the
223 subscales emotional problems, divided into negative mood/physical symptoms and negative
224 self-esteem, and functional problems divided into ineffectiveness and interpersonal problems.
225 A high score indicates more depressive symptoms. The questionnaire has demonstrated

For peer review only

excellent internal reliability both for 13-16 year olds ($\alpha = 0.89$), and for 17-21 year olds ($\alpha = 0.85$; 32). The scale has good test-retest reliability ($r = 0.60$; 32).

Recruitment

Participants are recruited through several channels. The first is through patient organisations. Patient organisations will be approached and asked if they are willing to advertise the Dutch YP Face IT study on their website and in their newsletters. If an adolescent is willing to participate, he/she is encouraged to send an e-mail to the research team, providing his/her contact details. The adolescent will then receive an information package containing a patient information letter, an informed consent form and a response card. The second channel is through our website (www.faceitvoorjongeren.nl). On our website, adolescents can read information about YP Face IT and contact the research team. If they do so, they will receive an information package. The third channel is through the Erasmus MC – Sophia Children's hospital. Subjects will be drawn from the department of Child and Adolescent Psychiatry/Psychology, the department of Plastic and Reconstructive Surgery, the department of Oral and Maxillofacial Surgery and Special Dental Care, and the department of Dermatology. Subjects will be informed about the study by their treating doctor/clinician. It will be explicitly stated that participation is voluntary and that they can withdraw from the study at any time should they wish to do so. After informing the potential participant, they will receive an information package. If a potential participant does not have regular appointments at the Erasmus MC – Sophia children's hospital, we will send an information package to their home address.

A response card will be included in the information package. On this response card, adolescents can indicate whether they give permission to be contacted by the research team. If

For peer review only

the response card has not been returned within two weeks, the research team will contact the adolescent.

If participants are willing to participate, they are asked to fill in a consent form. For participants younger than 16 years, both parents are required to sign the informed consent form. After receiving the informed consent form, the researchers will include the participant in the trial and send out the first set of online questionnaires.

Participants will receive a gift card worth €10,- after completing the entire study. If a participant decides to withdraw from the study prematurely, the adolescent will not receive a gift card.

Study design

A flow chart of the RCT is depicted in Figure 1. Prior to baseline, the in- and exclusion criteria as mentioned before (with the exception of appearance-related distress) are queried in a telephone call with the parent or adolescent. Appearance-related distress is measured based on the questionnaires completed at T1.

For peer review only

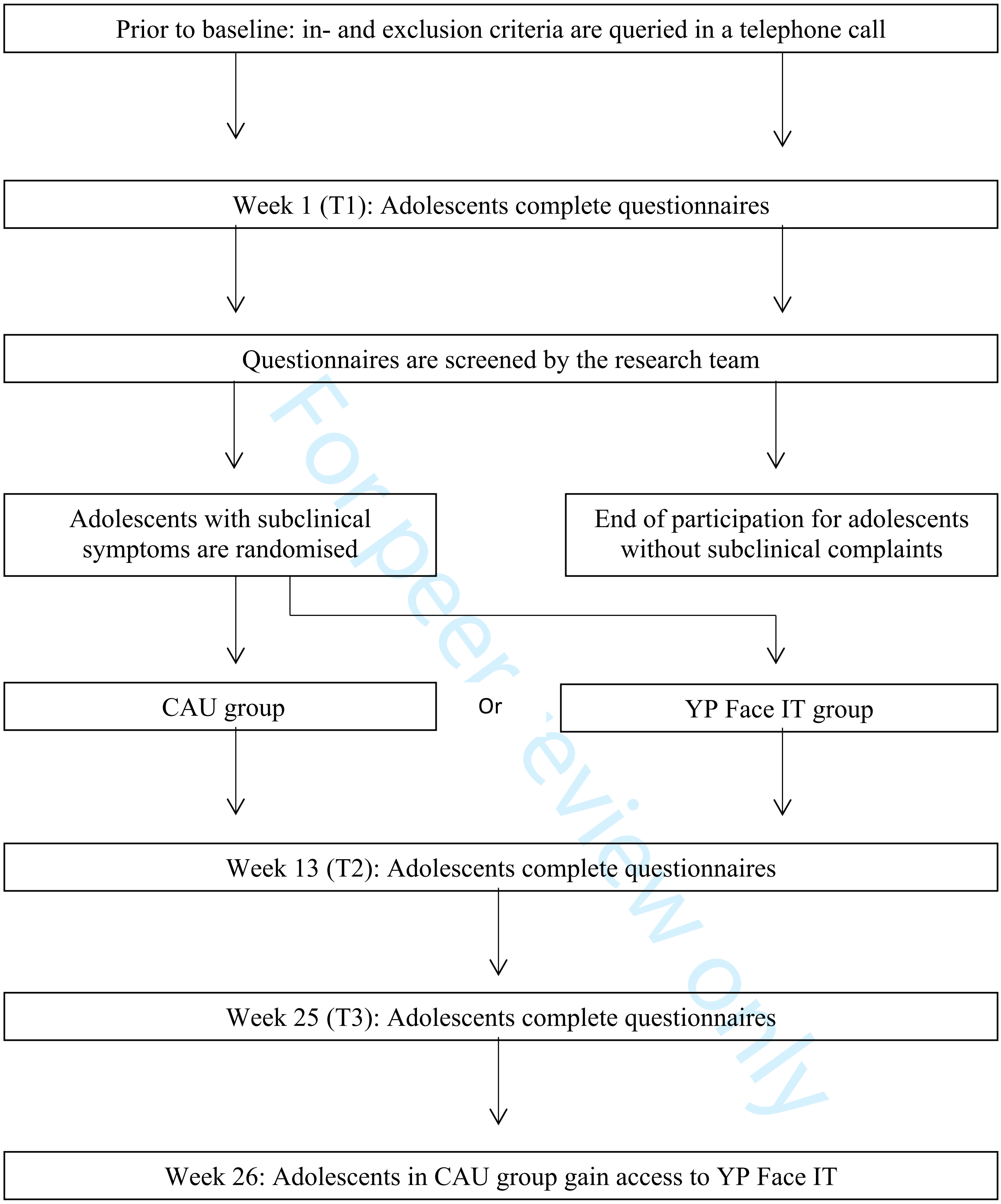

Figure 1. Flow chart of the study design

For peer review only

After completing the questionnaires, screening for appearance-related distress will take place by the research team. The scores on the SAS-A, BESAA and CDI-2 are considered. For the SAS-A and the BESAA adolescents that score 0.5-2.0 standard deviations above average will be included in the study. For the CDI-2 we will include adolescents scoring in the 70th to 90th percentile, as the manual states that this corresponds to subclinical symptoms of depression(32). Adolescents that score within the defined range for one or more of the questionnaires will be randomised into the study. Adolescents that do not score within these ranges will be excluded from participation.

Adolescents with subclinical symptoms will be randomised to either Care As Usual (CAU) or YP Face IT. Adolescents in the CAU group will receive CAU and will complete T2 at 13 weeks and T2 at 25 weeks. The CAU group will have the opportunity to complete YP Face IT after their participation in the study. Adolescents in the YP Face IT group will receive CAU and YP Face IT, and will complete questionnaires immediately after completing YP Face IT (T2; 13 weeks) and three months after completing YP Face IT (T3; 25 weeks).

Randomisation and blinding

Participants will be randomised to either the CAU or the YP Face IT group in a 1:1 ratio. Randomisation will be stratified by gender (girl or boy) and age (12-13, 14-15 or 16-17 years old). Randomisation will be done a list with random sequence, generated using a computer program. With the stratification, six different types of blocks can be formed. Each block will independently vary between four and eight randomisation pairs. The adolescents are informed of the group they are randomised to through e-mail. The researcher analysing the data will be blind to the randomisation and will not be involved in monitoring the adolescents in the intervention. The participants, the clinical psychologist and student monitoring the adolescents in the intervention will not be blind to the treatment conditions. When contacting

For peer review only

the research team, the adolescents are explicitly instructed not to talk about the group they were allocated to.

If, through monitoring, the research team suspects that a participant experiences clinically significant symptoms, unblinding may take place. The participant will be excluded from the study and will be transferred to more appropriate care.

Participants that drop out after randomisation will be asked if they are willing to complete the questionnaires at T2 and T3. They are in no way obliged to do so.

Sample size

For the sample size calculation we applied a mixed model ANOVA procedure, with a power of 0.80, a two-sided alpha of α=0.025 (Bonferroni corrected for two primary outcome measures), a correlation between the repeated measurements of r=0.70 and three repeated linearly decreasing measurements. Bessell and colleagues(17) reported effect sizes of d = 0.83 for appearance and d = 0.80 for anxiety in a study on Face IT. However, as we are not certain that we will obtain such a large effect, we will consider a medium effect of d = 0.50. To detect a medium effect, 56 participants are needed in both groups. Anticipating a drop-out of 50%, 224 participants are needed in total.

Statistical analysis

The data will be analysed using multilevel linear regression analyses. There will be two levels in the models. The participants constitute the upper level, their repeated measures the lower level. For each outcome variable a model will be postulated using treatment group, logarithm of time and interaction with treatment group as fixed effects. The logarithm of time is applied as generally treatments have the largest effect at start and the level of functioning stabilizes on the long run. The deviance statistic using restricted maximum likelihood will be

For peer review only

applied to determine the covariance structure. Effect sizes will be calculated from dividing differences between the primary time-point (i.e. three months) estimations and baseline by the estimated baseline standard deviation.

Participants who do not experience subclinical symptoms at T1, and are thus not randomised, will not be included in the analyses.

Monitoring

As the risks associated with this study are minimal, monitoring will take place once a year. An independent investigator will randomly check study documents, participant selection and participant safety. As minimal risks are involved, no interim analyses will be done.

Auditing may be done by the Erasmus MC – Sophia Children's hospital.

Patient and public involvement (PPI)

The original YP Face it intervention was developed in collaboration with adolescents and the charity Changing Faces(16). For the Dutch translation, 6 adolescents and their parents have read and reviewed the translations. Patient associations will be invited to help develop our dissemination strategy.

Trial status

The trial described in this paper has started in September 2019. Recruitment of participants will be until the July 2020. Data collection will be until January 2021, after which the data will be analysed.

ETHICS AND DISSEMINATION

Ethics, consent and permission

This study will be conducted in accordance to the Declaration of Helsinki. Research ethics approval was obtained from the Medical Ethics review Committee (MERC)

For peer review only

335 Rotterdam (reference number MEC-2018-052/NL63955.078.18). Any amendments to the
336 study protocol and (serious) adverse events will be reported to this committee.

337 **Data management and confidentiality**

338 To ensure anonymous processing of the data, every participant will receive a study
339 number. All research data will be processed using only this study number. The handling of
340 data will comply with the European General Data Protection Regulation (GDPR). The study
341 master file and all research data will be saved for 15 years, unless participants or their
342 parents/guardians do not give consent for saving their data.

343 All data will be stored in locked cabinets, or password-protected documents. The data,
344 including the final dataset, can be accessed only by members of the research team.

345 **Dissemination of research findings**

346 Results of this study will be presented on national and international conferences and in
347 peer-reviewed scientific journals, within the scope of the target groups. The results will also
348 be relayed to patients through patient organisations. Participants are also given the option to
349 sign up for a brief study report after the results are finalised. If deemed effective, YP Face IT
350 will be implemented in hospitals in The Netherlands.

351 **DISCUSSION**

352 The Dutch YP Face IT study aims to test the Dutch version of YP Face IT. With this
353 RCT we aim to retrieve data on the effectiveness of the intervention.

354 **Strengths and limitations**

355 A particular strength of this study is that it follows a stage model of behavioural
356 therapies research(33) and has excellent methodological quality. Previous research has lacked

For peer review only

methodological rigor, due to small sample sizes, not including a control group or not blinding personnel or participants(15). Due to the nature of this study, blinding of participants is not possible. However, this study does include an adequate sample size, includes a control group and blinding of relevant personnel. With this methodological quality, we hope to be able to provide evidence-base for YP Face IT and thus provide one of the first evidence-based treatments for adolescents with a visible difference(15). Furthermore, by recruiting our participants through several channels, we will be able to obtain a sample that is representative for Dutch adolescents in general rather than solely Rotterdam, where the research team is located. Because the intervention is available online, participants throughout the entire country are able to participate without adding extra burden to participating.

In addition to strengths, this study also has some limitations. First, the intervention is available for a range of conditions causing a visible difference. The overall group results might not be generalisable to one specific group. Second, participants are not blind to the treatment condition. Participants are informed about the randomisation groups. They are also informed that, if randomised to the CAU group, they will have the possibility to access YP Face IT after the study. This is a more ethical option than not offering treatment after the study and this knowledge may help keep attrition low. However, waiting list designs may lead to an overestimate of the treatment effect(34). Furthermore, this study includes only self-report measures. Diagnostic interviews or parent-reports could provide more enriched data. However, due to time and financial constraints these data will not be collected.

For peer review only

Reference list

1. Changing Faces. Prevalence of disfigurement 2010 [Available from: http://admin.changingfaces.org.uk/downloads/FE%20Campaign,%20Epidemiology%202%20pages.pdf.

2. Berger ZE, Dalton LJ. Coping with a cleft: psychosocial adjustment of adolescents with a cleft lip and palate and their parents. The Cleft Palate-Craniofacial Journal. 2009;46(4):435-43. doi:10.1597/08-093.1

3. Díaz-Atienza F, Gurpegui M. Environmental stress but not subjective distress in children or adolescents with alopecia areata. Journal of Psychosomatic Research. 2011;71(2):102-7. doi:10.1016/j.jpsychores.2011.01.007

4. Feragen KB, Kvalem IL, Rumsey N, Borge AIH. Adolescents with and without a facial difference: The role of friendships and social acceptance in perceptions of appearance and emotional resilience. Body Image. 2010;7(4):271-9. doi:10.1016/j.bodyim.2010.05.002

5. Thompson A, Kent G. Adjusting to disfigurement: processes involved in dealing with being visibly different. Clinical Psychology review. 2001;21(5):663-82. doi:10.1016/S0272-7358(00)00056-8

6. Rumsey N, Harcourt D. Visible difference amongst children and adolescents: issues and interventions. Developmental Neurorehabilitation. 2007;10(2):113-23. doi:10.1080/13638490701217396

7. Barankin B, DeKoven J. Psychosocial effect of common skin diseases. Canadian Family Physician. 2002;48:712-6.

8. Magin PJ, Pond CD, Smith WT, Watson AB, Goode SM. A cross-sectional study of psychological morbidity in patients with acne, psoriasis and atopic dermatitis in specialist dermatology and general practices. Journal of the European Academy of Dermatology and Venereology. 2008;22(12):1435-44. doi:10.1111/j.1468-3083.2008.02890.x

9. Pope AW, Snyder HT. Psychosocial Adjustment in Children and Adolescents With a Craniofacial Anomaly: Age and Sex Patterns. The Cleft Palate-Craniofacial Journal. 2005;42(4):349-54. doi:10.1597/04-043r.1

10. Bakker A, Maertens KJP, Van Son MJM, Van Loey NEE. Psychological consequences of pediatric burns from a child and family perspective: A review of the empirical literature. Clinical Psychology Review. 2013;33(3):361-71. doi:10.1016/j.cpr.2012.12.006

11. Turner SR, Rumsey N, Sandy JR. Psychological aspects of cleft lip and palate. European Journal of Orthodontics. 1998;20(4):407-15. doi:10.1093/ejo/20.4.407

12. Bessell A, Moss TP. Evaluating the effectiveness of psychosocial interventions for individuals with visible differences: a systematic review of the empirical literature. Body Image. 2007;4(3):227-38. doi:10.1016/j.bodyim.2007.04.005

13. Muftin Z, Thompson AR. A systematic review of self-help for disfigurement: effectiveness, usability, and acceptability. Body Image. 2013;10(4):442-50. doi:10.1016/j.bodyim.2013.07.005

14. Norman A, Moss TP. Psychosocial interventions for adults with visible differences: a systematic review. PeerJ. 2015;3:e870. doi:10.7717/peerj.870

15. Jenkinson E, Williamson H, Byron-Daniel J, Moss TP. Systematic Review: Psychosocial Interventions for Children and Young People With Visible Differences Resulting From Appearance Altering Conditions, Injury, or Treatment Effects. Journal of Pediatric Psychology. 2015;40(10):1017-33. doi:10.1093/jpepsy/jsv048

16. Williamson H, Griffiths C, Harcourt D. Developing young person's Face IT: Online psychosocial support for adolescents struggling with conditions or injuries affecting their appearance. Health Psychology Open. 2015;2(2):1-12. doi:10.1177/2055102915619092

17. Bessell A, Brough V, Clarke A, Harcourt D, Moss TP, Rumsey N. Evaluation of the effectiveness of Face IT, a computer-based psychosocial intervention for disfigurement-related distress. Psychology, Health & Medicine. 2012;17(5):565-77. doi:10.1080/13548506.2011.647701

For peer review only - http://bmjopen.bmj.com/site/about/guidelines.xhtml

426 18. La Greca AM, Lopez N. Social anxiety among adolescents: Linkages with peer relations and friendships. Journal of Abnormal Child Psychology. 1998;26(2):83-94. doi:10.1023/A:1022684520514
428 19. Inderbitzen-Nolan HM, Walters KS. Social anxiety scale for adolescents: Normative data and further evidence of construct validity. Journal of Clinical Child Psychology. 2000;29(3):360-71. doi:10.1207/S15374424JCCP2903_7
431 20. Mendelson BK, Mendelson MJ, White DR. Body-esteem scale for adolescents and adults. Journal of Personality Assessment. 2001;76(1):90-106. doi:10.1207/S15327752JPA7601_6
433 21. Cragun D, DeBate RD, Ata RN, Thompson JK. Psychometric properties of the Body Esteem Scale for Adolescents and Adults in an early adolescent sample. Eating and Weight Disorders. 2013;18(3):275-82. doi:10.1007/s40519-013-0031-1
436 22. Haubrock I. Body-self-unity: Gender difference concerning the body self unity. Do the genders differ in the evaluation of their self and body? [Bachelor thesis]. Enschede: University of Twente; 2009.
439 23. Harter S. Self-perception profile for adolescents: Manual and questionnaires. In: Denver Uo, editor. Denver2012.
441 24. Treffers PDA, Goedhart AW, Veerman JW, Van den Bergh BRH, Ackaert L, De Rycke L. Competentie belevingsschaal voor Adolescenten. Tijdschrift voor Psychiatrie. 2004;7:468-9.
443 25. Lawrence JW, Fauerbach JA, Heinberg LJ, Doctor M, Thombs BD. The reliability and validity of the Perceived Stigmatization Questionnaire (PSQ) and the Social Comfort Questionnaire (SCQ) among an adult burn survivor sample. Psychological Assessment. 2006;18(1):106-11. doi:10.1037/1040-3590.18.1.106
447 26. Lawrence JW, Rosenberg L, Rimmer RB, Thombs BD, Fauerbach JA. Perceived stigmatization and social comfort: validating the constructs and their measurement among pediatric burn survivors. Rehabilitation Psychology. 2010;55(4):360-71. doi:10.1037/a0021674
450 27. Masnari O, Schiestl C, Rossler J, Gutlein SK, Neuhaus K, Weibel L, et al. Stigmatization predicts psychological adjustment and quality of life in children and adolescents with a facial difference. Journal of Pediatric Psychology. 2013;38(2):162-72. doi:10.1093/jpepsy/jss106
453 28. Herdman M, Gudex C, Lloyd A, Janssen MF, Kind P, Parkin D, et al. Development and preliminary testing of the new five-level version of EQ-5D (EQ-5D-5L). Quality of Life Research. 2011;20(10):1727-36. doi:10.1007/s11136-011-9903-x
456 29. Janssen MF, Pickard AS, Golicki D, Gudex C, Niewada M, Scalone L, et al. Measurement properties of the EQ-5D-5L compared to the EQ-5D-3L across eight patient groups: a multi-country study. Quality of Life Research. 2013;22(7):1717-27. doi:10.1007/s11136-012-0322-4
459 30. Diedrichs PC, Atkinson MJ, Steer RJ, Garbett KM, Rumsey N, Halliwell E. Effectiveness of a brief school-based body image intervention 'Dove Confident Me: Single Session'when delivered by teachers and researchers: Results from a cluster randomised controlled trial. Behaviour Research and Therapy. 2015;74:94-104. doi:10.1016/j.brat.2015.09.004
463 31. Okkerse JME, Dessens AB. Spiegeltje, spiegeltje.. Vragenlijst voor adolescenten en volwassenen over (on)tevredenheid met het lichaam en gerelateerde lijdensdruk. 2016.
465 32. Kovacs M. CDI-2. Screeningsvragenlijst voor depressie bij kinderen en jongeren. Nederlandse bewerking door Denise Bodden CBeYS, editor. Amsterdam: Hogrefe Uitgevers BV; 2016.
467 33. Rounsaville BJ, Carroll KM, Onken LS. A Stage Model of Behavioral Therapies Research: Getting Started and Moving on From Stage I. Clinical Psychology: Science and Practice. 2001;8(2):133-42. doi:10.1093/clipsy.8.2.133
470 34. Cunningham JA, Kypri K, McCambridge J. Exploratory randomized controlled trial evaluating the impact of a waiting list control design. BMC Medical Research Methodology. 2013;13(1):150. doi:10.1186/1471-2288-13-150

473

474

For peer review only

DECLARATIONS

Author statement. JO, HW and EU developed the study design. RT made the statistical analysis plan for the study. JO initiated the study and obtained funding. JO and EU supervise the study and data collection. MvD conducts the data collection, along with psychology and medical students. MvD wrote the first draft of the manuscript. All authors read and approved the final manuscript.

Funding. This work was supported by a grant from the Innovatiefonds Zorgverzekeraars [grant number B 17-133/Dossier 3446]. The funding body has no role in the design of the study and the collection, analysis and interpretation of the data during this study.

Availability of data and material. Data sharing is not applicable to this article as no new data were created or analysed in this manuscript.

Competing interests. None declared.

Acknowledgements. Not applicable.

For peer review only

For peer review only - http://bmjopen.bmj.com/site/about/guidelines.xhtml

STANDARD PROTOCOL ITEMS: RECOMMENDATIONS FOR INTERVENTIONAL TRIALS

SPIRIT 2013 Checklist: Recommended items to address in a clinical trial protocol and related documents*

Section/item	Item No	Description	Addressed on page number
**Administrative information**			
Title	1	Descriptive title identifying the study design, population, interventions, and, if applicable, trial acronym	1
Trial registration	2a	Trial identifier and registry name. If not yet registered, name of intended registry	2
	2b	All items from the World Health Organization Trial Registration Data Set	In trial registry
Protocol version	3	Date and version identifier	page header
Funding	4	Sources and types of financial, material, and other support	21
Roles and responsibilities	5a	Names, affiliations, and roles of protocol contributors	1, 21
	5b	Name and contact information for the trial sponsor	21
	5c	Role of study sponsor and funders, if any, in study design; collection, management, analysis, and interpretation of data; writing of the report; and the decision to submit the report for publication, including whether they will have ultimate authority over any of these activities	21
	5d	Composition, roles, and responsibilities of the coordinating centre, steering committee, endpoint adjudication committee, data management team, and other individuals or groups overseeing the trial, if applicable (see Item 21a for data monitoring committee)	n/a

For peer review only

**Introduction**			
Background and rationale	6a	Description of research question and justification for undertaking the trial, including summary of relevant studies (published and unpublished) examining benefits and harms for each intervention	4,5
	6b	Explanation for choice of comparators	7
Objectives	7	Specific objectives or hypotheses	5
Trial design	8	Description of trial design including type of trial (eg, parallel group, crossover, factorial, single group), allocation ratio, and framework (eg, superiority, equivalence, noninferiority, exploratory)	12-15
**Methods: Participants, interventions, and outcomes**			
Study setting	9	Description of study settings (eg, community clinic, academic hospital) and list of countries where data will be collected. Reference to where list of study sites can be obtained	6,11
Eligibility criteria	10	Inclusion and exclusion criteria for participants. If applicable, eligibility criteria for study centres and individuals who will perform the interventions (eg, surgeons, psychotherapists)	6
Interventions	11a	Interventions for each group with sufficient detail to allow replication, including how and when they will be administered	6,7
	11b	Criteria for discontinuing or modifying allocated interventions for a given trial participant (eg, drug dose change in response to harms, participant request, or improving/worsening disease)	7
	11c	Strategies to improve adherence to intervention protocols, and any procedures for monitoring adherence (eg, drug tablet return, laboratory tests)	7
	11d	Relevant concomitant care and interventions that are permitted or prohibited during the trial	7
Outcomes	12	Primary, secondary, and other outcomes, including the specific measurement variable (eg, systolic blood pressure), analysis metric (eg, change from baseline, final value, time to event), method of aggregation (eg, median, proportion), and time point for each outcome. Explanation of the clinical relevance of chosen efficacy and harm outcomes is strongly recommended	8-10
Participant timeline	13	Time schedule of enrolment, interventions (including any run-ins and washouts), assessments, and visits for participants. A schematic diagram is highly recommended (see Figure)	11-14

For peer review only - http://bmjopen.bmj.com/site/about/guidelines.xhtml

Sample size	14	Estimated number of participants needed to achieve study objectives and how it was determined, including clinical and statistical assumptions supporting any sample size calculations	15
Recruitment	15	Strategies for achieving adequate participant enrolment to reach target sample size	11,10

Methods: Assignment of interventions (for controlled trials)

Allocation:

Sequence generation	16a	Method of generating the allocation sequence (eg, computer-generated random numbers), and list of any factors for stratification. To reduce predictability of a random sequence, details of any planned restriction (eg, blocking) should be provided in a separate document that is unavailable to those who enrol participants or assign interventions	14,15
Allocation concealment mechanism	16b	Mechanism of implementing the allocation sequence (eg, central telephone; sequentially numbered, opaque, sealed envelopes), describing any steps to conceal the sequence until interventions are assigned	14,15
Implementation	16c	Who will generate the allocation sequence, who will enrol participants, and who will assign participants to interventions	14,15
Blinding (masking)	17a	Who will be blinded after assignment to interventions (eg, trial participants, care providers, outcome assessors, data analysts), and how	14,15
	17b	If blinded, circumstances under which unblinding is permissible, and procedure for revealing a participant's allocated intervention during the trial	14,15

Methods: Data collection, management, and analysis

Data collection methods	18a	Plans for assessment and collection of outcome, baseline, and other trial data, including any related processes to promote data quality (eg, duplicate measurements, training of assessors) and a description of study instruments (eg, questionnaires, laboratory tests) along with their reliability and validity, if known. Reference to where data collection forms can be found, if not in the protocol	8-10
	18b	Plans to promote participant retention and complete follow-up, including list of any outcome data to be collected for participants who discontinue or deviate from intervention protocols	15

Data management	19	Plans for data entry, coding, security, and storage, including any related processes to promote data quality (eg, double data entry; range checks for data values). Reference to where details of data management procedures can be found, if not in the protocol	17
Statistical methods	20a	Statistical methods for analysing primary and secondary outcomes. Reference to where other details of the statistical analysis plan can be found, if not in the protocol	15,16
	20b	Methods for any additional analyses (eg, subgroup and adjusted analyses)	15,16
	20c	Definition of analysis population relating to protocol non-adherence (eg, as randomised analysis), and any statistical methods to handle missing data (eg, multiple imputation)	15,16
**Methods: Monitoring**			
Data monitoring	21a	Composition of data monitoring committee (DMC); summary of its role and reporting structure; statement of whether it is independent from the sponsor and competing interests; and reference to where further details about its charter can be found, if not in the protocol. Alternatively, an explanation of why a DMC is not needed	16
	21b	Description of any interim analyses and stopping guidelines, including who will have access to these interim results and make the final decision to terminate the trial	15,16
Harms	22	Plans for collecting, assessing, reporting, and managing solicited and spontaneously reported adverse events and other unintended effects of trial interventions or trial conduct	16,17
Auditing	23	Frequency and procedures for auditing trial conduct, if any, and whether the process will be independent from investigators and the sponsor	16
**Ethics and dissemination**			
Research ethics approval	24	Plans for seeking research ethics committee/institutional review board (REC/IRB) approval	16,17
Protocol amendments	25	Plans for communicating important protocol modifications (eg, changes to eligibility criteria, outcomes, analyses) to relevant parties (eg, investigators, REC/IRBs, trial participants, trial registries, journals, regulators)	16,17

For peer review only - http://bmjopen.bmj.com/site/about/guidelines.xhtml

Consent or assent	26a	Who will obtain informed consent or assent from potential trial participants or authorised surrogates, and how (see Item 32)	11,12
	26b	Additional consent provisions for collection and use of participant data and biological specimens in ancillary studies, if applicable	n/a
Confidentiality	27	How personal information about potential and enrolled participants will be collected, shared, and maintained in order to protect confidentiality before, during, and after the trial	17
Declaration of interests	28	Financial and other competing interests for principal investigators for the overall trial and each study site	21
Access to data	29	Statement of who will have access to the final trial dataset, and disclosure of contractual agreements that limit such access for investigators	17
Ancillary and post-trial care	30	Provisions, if any, for ancillary and post-trial care, and for compensation to those who suffer harm from trial participation	n/a
Dissemination policy	31a	Plans for investigators and sponsor to communicate trial results to participants, healthcare professionals, the public, and other relevant groups (eg, via publication, reporting in results databases, or other data sharing arrangements), including any publication restrictions	17
	31b	Authorship eligibility guidelines and any intended use of professional writers	n/a
	31c	Plans, if any, for granting public access to the full protocol, participant-level dataset, and statistical code	n/a
**Appendices**			
Informed consent materials	32	Model consent form and other related documentation given to participants and authorised surrogates	In appendix
Biological specimens	33	Plans for collection, laboratory evaluation, and storage of biological specimens for genetic or molecular analysis in the current trial and for future use in ancillary studies, if applicable	n/a

*It is strongly recommended that this checklist be read in conjunction with the SPIRIT 2013 Explanation & Elaboration for important clarification on the items. Amendments to the protocol should be tracked and dated. The SPIRIT checklist is copyrighted by the SPIRIT Group under the Creative Commons "Attribution-NonCommercial-NoDerivs 3.0 Unported" license.

For peer review only - http://bmjopen.bmj.com/site/about/guidelines.xhtml

BMJ Open

The Dutch YP Face IT Study, investigating online psychological treatment for Adolescents with a Visible Difference: Study Protocol of a Randomised Controlled Trial

Journal:	*BMJ Open*
Manuscript ID	bmjopen-2020-041449.R1
Article Type:	Protocol
Date Submitted by the Author:	14-Dec-2020
Complete List of Authors:	van Dalen, Marije; Erasmus MC Sophia Children Hospital, Department of Child and Adolescent Psychiatry/Psychology<br>Pasmans, Suzanne G. M. A.; Erasmus MC Sophia Children Hospital, Department of Dermatology<br>Aendekerk, Marie-Louise; Erasmus MC Sophia Children Hospital, Department of Child and Adolescent Psychiatry/Psychology<br>Mathijssen, Irene; Erasmus MC Sophia Children Hospital, Department of Plastic and Reconstructive Surgery<br>Koudstaal, Maarten; Erasmus MC Sophia Children Hospital, Department of Oral and Maxillofacial Surgery<br>Timman, Reinier; Erasmus MC, Department of Psychiatry, Section of Medical Psychology & Psychotherapy<br>Williamson, Heidi; University of the West of England, Health and Life Sciences<br>Hillegers, Manon; Erasmus MC Sophia Children Hospital, Department of Child and Adolescent Psychiatry/Psychology<br>Utens, Elisabeth; Erasmus MC Sophia Children Hospital, Department of Child and Adolescent Psychiatry/Psychology<br>Okkerse, Jolanda; Erasmus MC Sophia Children Hospital, Department of Child and Adolescent Psychiatry/Psychology
**Primary Subject Heading**:	Mental health
Secondary Subject Heading:	Dermatology, Surgery, Paediatrics
Keywords:	Child & adolescent psychiatry < PSYCHIATRY, Anxiety disorders < PSYCHIATRY, Depression & mood disorders < PSYCHIATRY

SCHOLARONE™
Manuscripts

I, the Submitting Author has the right to grant and does grant on behalf of all authors of the Work (as defined in the below author licence), an exclusive licence and/or a non-exclusive licence for contributions from authors who are: i) UK Crown employees; ii) where BMJ has agreed a CC-BY licence shall apply, and/or iii) in accordance with the terms applicable for US Federal Government officers or employees acting as part of their official duties; on a worldwide, perpetual, irrevocable, royalty-free basis to BMJ Publishing Group Ltd ("BMJ") its licensees and where the relevant Journal is co-owned by BMJ to the co-owners of the Journal, to publish the Work in this journal and any other BMJ products and to exploit all rights, as set out in our licence.

The Submitting Author accepts and understands that any supply made under these terms is made by BMJ to the Submitting Author unless you are acting as an employee on behalf of your employer or a postgraduate student of an affiliated institution which is paying any applicable article publishing charge ("APC") for Open Access articles. Where the Submitting Author wishes to make the Work available on an Open Access basis (and intends to pay the relevant APC), the terms of reuse of such Open Access shall be governed by a Creative Commons licence – details of these licences and which Creative Commons licence will apply to this Work are set out in our licence referred to above.

Other than as permitted in any relevant BMJ Author's Self Archiving Policies, I confirm this Work has not been accepted for publication elsewhere, is not being considered for publication elsewhere and does not duplicate material already published. I confirm all authors consent to publication of this Work and authorise the granting of this licence.

For peer review only

The Dutch YP Face IT Study, investigating online psychological treatment for Adolescents with a Visible Difference: Study Protocol of a Randomised Controlled Trial

M. van Dalen[1], S.G.M.A. Pasmans[2], E.W.C. Aendekerk[1], I.M.J. Mathijssen[3], M.J. Koudstaal[4], R. Timman[5], H. Williamson[6], M.H.J. Hillegers[1], E.M.W.J. Utens[1,7,8], J.M.E. Okkerse[1]

1. Department of Child and Adolescent Psychiatry/Psychology, Erasmus MC-Sophia Children's Hospital, Rotterdam, The Netherlands. 2. Department of Dermatology, Centre of Pediatric Dermatology, Erasmus MC-Sophia Children's Hospital, Rotterdam, The Netherlands. 3. Department of Plastic and Reconstructive Surgery and Hand Surgery, The Dutch Craniofacial Centre, Erasmus MC-Sophia Children's Hospital, Rotterdam, The Netherlands. 4. Department of Oral and Maxillofacial Surgery, The Dutch Craniofacial Centre, Erasmus MC-Sophia Children's Hospital, Rotterdam, The Netherlands. 5. Department of Psychiatry, unit of Medical Psychology and Psychotherapy, Erasmus MC, Rotterdam, The Netherlands. 6. Department of Health and Social Sciences, University of the West of England, Bristol, United Kingdom 7.Research Institute of Child Development and Education, University of Amsterdam, Amsterdam, The Netherlands. 8. Academic Center for Child Psychiatry the Bascule/ Department of Child and Adolescent Psychiatry, Amsterdam University Medical Center, Amsterdam, The Netherlands.

Corresponding author:

Dr. Jolanda Okkerse

Department of Child and Adolescent Psychiatry/Psychology

Erasmus MC-Sophia Children's Hospital, P.O. Box 2060,

3000 CB Rotterdam, The Netherlands

E-mail: j.okkerse@erasmusmc.nl

Tel.: 0031 (0) 10 704 02 09

Word count (excluding title page, abstract, references, figures and tables, max. 4000): 3995

For peer review only

Author e-mail addresses:

Marije van Dalen m.vandalen.1@erasmusmc.nl

Suzanne G.M.A. Pasmans s.pasmans@erasmusmc.nl

E.W.C. (Marie-Louise) Aendekerk e.aendekerk@erasmusmc.nl

Irene M.J. Mathijssen i.mathijssen@erasmusmc.nl

Maarten J. Koudstaal m.koudstaal@erasmusmc.nl

Reinier Timman r.timman@erasmusmc.nl

Heidi Williamson heidi3.williamson@uwe.ac.uk

Manon H.J. Hillegers m.hillegers@erasmusmc.nl

Elisabeth M.W.J. Utens l.utens@debascule.com

Jolanda M.E. Okkerse j.okkerse@erasmusmc.nl

For peer review only

Abstract

Introduction: This paper outlines the study protocol for the Dutch YP Face IT study. Adolescents with a visible difference (i.e. disfigurement) often experience challenging social situations such as being stared at, receiving unwanted questions or being teased. As a consequence, some of these adolescents experience adverse psychosocial outcomes and appearance-related distress. To address this appearance-related distress, an online psychotherapeutic intervention, YP Face IT, has been developed. YP Face IT uses Social Skills Interaction Training and Cognitive Behavioural Therapy. The Dutch YP Face IT study tests whether this intervention is effective in reducing social anxiety and improving body esteem. **Methods and analysis:** Participants are adolescents aged 12-18 with a visible difference and experiencing appearance-related distress. In this two-armed Randomised Controlled Trial, 224 adolescents will be randomised to care as usual or YP Face IT. Adolescents will complete questionnaires at baseline, at 13 weeks and at 25 weeks. Primary outcomes are differences in social anxiety and body esteem between YP Face IT and care as usual. Secondary outcomes are differences in aspects of self-worth, perceived stigmatisation, health-related quality of life, life engagement, appearance-related distress and depressive symptoms between the two groups. **Ethics and dissemination:** Research ethics approval was obtained from the Medical Ethics review Committee Rotterdam (reference number MEC-2018-052/NL63955.078.18). Findings will be disseminated through academic peer-reviewed publications, conferences and in newsletters to patient associations and participants of the study. **Trial registration:** This trial was registered in The Netherlands trial register, NL7626, Registered 26th March 2019, https://www.trialregister.nl/trial/7626.

Keywords: Visible difference; appearance-related distress; adolescents; anxiety; body-esteem

For peer review only

ARTICLE SUMMARY

Strengths and limitations of this study

- This is the first Randomised Controlled Trial (RCT) to assess YP Face IT, an online psychosocial intervention for adolescents with a visible difference.
- Measurements include a direct follow-up and follow-up three months after completion of the intervention.
- Participants are recruited throughout The Netherlands, enhancing generalisability.
- Participants are not blinded to allocation, which could lead to reporting bias.

INTRODUCTION

Having a visible difference can have a substantial impact on one's life. People with a visible difference commonly encounter difficult social situations, such as being stared at, receiving unwanted questions and feeling isolated. A visible difference (i.e. disfigurement) to the face or body occurs in 1 in 44 people, a visible difference solely to the facial area in 1 in 111(1). A visible difference can be either congenital (e.g. cleft lip and palate, craniofacial conditions), result from a skin condition (e.g. vitiligo, psoriasis and acne), trauma (e.g. burns, scars), disease (e.g. cancer, meningitis and alopecia areata) or medical treatment (e.g. surgery or radiotherapy). Whether someone identifies as having a visible difference is a subjective experience. A clear definition of what a visible difference is can thus not be given.

Some studies show that adolescents with a visible difference experience no additional psychosocial problems(2, 3) and even perceive their friendship, social acceptance, appearance and emotional distress in a more positive way than unaffected peers(4). However, reviews show that a significant amount of adolescents with a visible difference do experience adverse psychosocial outcomes(5-7). More specifically, they experience higher levels of minor

For peer review only

psychological disturbance, self-consciousness in public situations, neuroticism(8), internalising symptoms(7, 9) and anxiety, depression and difficulties with social functioning(10). Furthermore, parents perceive their children as being more shy and inhibited(11).

Available interventions for adolescents or adults with a visible difference are scarce and lack evidence-base(12-15). Research often lacks a solid design and overall methodological quality, and yields small effect sizes. However, there is some support for models involving Social Skills Training (SST), Cognitive Behavioural Therapy (CBT; 12, 14, 15) and self-help for managing anxiety(13).

YP (Young People) Face IT was developed in the UK, in collaboration with adolescents and the charity Changing Faces(16). YP Face IT was derived from the adult intervention Face IT which was found to be effective at reducing anxiety, depression and appearance concerns whilst increasing positive adjustment(17). In an acceptability and feasibility study concerning YP Face IT, the intervention was found to be acceptable, with most of the adolescents thinking it would help them improve their confidence and self-acceptance and help them develop new skills in dealing with difficult social situations(16).

Aims

In sum, interventions aimed at adolescents with a visible difference are scarce and have limited evidence to support them. The available evidence, however, does point to SST and CBT as being potentially effective methods for managing psychosocial difficulties amongst people with a visible difference. In an attempt to provide adolescents coping with a visible difference with an effective intervention focussing on improving emotional resilience, the Dutch YP Face IT study aims to test an online psychotherapeutic intervention combining Social Interaction Skills Training (SIST) and CBT. More specifically, the aims of the current

For peer review only

study are to test the effectiveness of the Dutch version of YP Face IT in reducing anxiety and improving body-esteem amongst Dutch adolescents with a visible difference.

METHODS AND ANALYSIS

To assess the effectiveness of the Dutch version of YP Face IT, a randomised controlled trial (RCT) will be conducted. This trial was registered prospectively in the Netherlands Trial Register (NL7626).

Participants

This study will be conducted in a Dutch academic hospital. The target group for this study is Dutch adolescents aged 12-18 with a visible difference and experiencing appearance-related distress. Recruitment will take place throughout The Netherlands.

Adolescents will be eligible for participation if they are aged between 12 and 18, have a visible difference, access to a computer with internet and experience appearance-related distress. The appearance-related distress is operationalised as an elevated score on either social anxiety or depression, or a lower score on body esteem, as assessed by self-reports.

Exclusion criteria are a mental disability, reading ability below 12 years of age, visual impairments preventing comprehension of the online intervention, a clinical diagnosis of depression, psychosis, body dysmorphic disorder or an eating disorder, and insufficient proficiency of Dutch. Furthermore, adolescents who receive face-to-face care by a psychologist will be excluded from participation.

Intervention

YP Face IT (Dutch: *Face IT voor jongeren*) is an online psychotherapeutic intervention(16) specifically designed for adolescents and developed in cooperation with

For peer review only

adolescents and the British charity Changing Faces. The intervention is aimed at helping adolescents cope with some key concerns people with a visible difference may have, such as anxiety, depression, low self-esteem and appearance-related distress.

YP Face IT combines SIST with CBT. The intervention consists of seven weekly sessions and an additional booster session after six weeks, helping the adolescent recall what was learned during the weekly sessions. The first three sessions focus on SIST, sessions 4-6 focus on CBT and session 7 is a summary of all learned techniques. Session 8 is a booster quiz, six weeks after session 7. The content of each session is displayed in Table 1. Each session has different exercises and activities and takes 45-60 minutes to complete. In addition to the weekly sessions, participants will also be asked to complete small homework assignments between sessions. Participants will have access to an online journal in which they can write about their experiences and enter the answers to the homework assignments. Participants will have the opportunity to contact the research team if they are struggling with the homework activities. A reminder e-mail is send if the participant has not entered data relating to the homework activity in their journal within five days following their last session.

YP Face IT is designed as a self-help intervention, without any active guidance by a psychologist. Adolescents work through the sessions on their own. Parents receive a short description of each session and will receive the reminders, but are not involved in the sessions themselves.

Monitoring will take place to assess whether adolescents complete the homework assignments and whether they are struggling in any way with the intervention or their mental health. Monitoring will be done by an experienced clinical psychologist and a master's student. Should we find that adolescents experience severe psychological symptoms, they are referred to other care that may better suit their needs.

For peer review only

Table 1.

Content of the YP Face IT intervention

Session	Content	Type	Week
1. Common problems	Common problems experienced by adolescents with a visible difference; bystander responses, teasing, bullying.	SIST	1
2. Improve your social skills	Body language, verbal and non-verbal skills and building positive social skills.	SIST	2
3. Don't be SCARED, REACH OUT	Effect of own behavior on other people. Toolbox of techniques to cope with difficult social situations.	SIST	3
4. Think, Feel, Do	Relationship between thoughts, feelings and behaviours. Identifying negative thoughts and using a positive voice to challenges these thoughts. Different coping strategies for negative thoughts.	CBT	4
5. SMART goals	Using SMART goals to help achieve goals, dividing the end goal into sub goals. Dealing with concerns in romantic relationships.	CBT	5
6. Beating anxiety	Exposure therapy in the form of a fear ladder. Relaxation techniques.	CBT	6
7. Looking at your progress	Summary session, with summary of all previous sessions.	SIST/CBT	7
8. Booster quiz	Quiz with 16 questions on all taught techniques.	SIST/CBT	13

Note: SIST = Social Skills Interaction Training; CBT = Cognitive Behavioural Therapy

For peer review only

161 Care As Usual (CAU).

162 CAU will consist of regular medical care. The type of the care may differ depending
163 on the condition (e.g. skin care for eczema, orthodontics for cleft palate). Psychological care
164 is not part of CAU. However, medical personnel will monitor the adolescents' well-being as
165 part of CAU. They may refer the adolescents to a psychologist in case of suspicions of
166 psychological problems. Adolescents that receive psychological care during participation in
167 the study will not be allowed further participation.

168 **Materials**

169 All outcome measures are completed at all three time points.

170 Primary outcomes

171 *Social anxiety.* The Social Anxiety Scale for Adolescents (SAS-A; 18) will be used to
172 assess social anxiety. The SAS-A contains 22 statements that are rated on a 5-point Likert
173 scale (e.g. I worry about what others say about me; I get nervous when I meet new people). A
174 high score indicates high levels of social anxiety. The SAS-A contains three subscales,
175 namely Fear of Negative Evaluation (FNE), social avoidance and distress in new situations
176 (SAD – new), and social avoidance and distress in general (SAD-general). Cronbach's alphas
177 are satisfactory for the subscales, being $\alpha = 0.91$, 0.83 and 0.76 respectively (18). Further
178 research also yielded satisfactory Cronbach's alpha's of $\alpha = 0.89$, 0.80 and 0.70 for FNE,
179 SAD-new and SAD-general respectively (19).

180 *Body esteem.* The subscale 'appearance' from the Body Esteem Scale For Adolescents
181 and Adults (BESAA; 20) is used to assess participants attitudes and feelings about their
182 appearance. A higher score indicates more satisfaction with one's appearance. This subscale
183 consists of 10 statements, such as "I like how I look in photos". The items are rated on a 5-

For peer review only

184 point Likert scale ranging from “never” to “always”. The subscale demonstrates excellent
185 internal consistency in a sample of American girls (α = 0.92) and boys (α = 0.90; 21) and in a
186 sample of Dutch university students (α = 0.90; 22).

187 Secondary outcomes

188 *Aspects of self-worth.* The Dutch version of the Self-Perception Profiles for
189 Adolescents (SPPA; 23), the Competentiebelevingsschaal voor Adolescenten (CBSA; 24),
190 will be used to assess different aspects of self-worth. The CBSA consists of eight specific
191 domains and a global self-worth subscale. The specific domains are: scholastic competence,
192 social competence, athletic competence, physical appearance, job competence, romantic
193 appeal, behavioral conduct and close friendship. The CBSA consists of 35 items, each
194 consisting of two contrasting statements (e.g. some teenagers are *not* happy with the way they
195 look BUT other teenagers *are* happy with the way they look). The adolescent is asked to pick
196 the statement that is most representative of them and choose whether the statement is ‘sort of
197 true for me’ or ‘really true for me’. All domains demonstrate good to excellent internal
198 reliabilities, ranging from α = 0.74 to α = 0.93. The global self-worth subscale also
199 demonstrates excellent reliability, with α = 0.80-0.89(23).

200 *Perceived stigmatisation.* The Perceived Stigmatization Questionnaire (PSQ; 25) will
201 be used to measure stigmatisation behaviours commonly experienced by people with a visible
202 difference. The PSQ is divided into three subscales, namely: absence of friendly behaviour,
203 confused/staring behaviour and hostile behaviour. These subscales amount to a total of 21
204 items concerning stigmatising behaviours, such as “people avoid looking at me” and “people
205 call me names”. Participants are asked to rate how often they experience certain behaviour on
206 a 5-point Likert scale. A high score reflects high perceived stigmatisation. The three subscales

For peer review only

have been established(26) and the PSQ has adequate reliability in a sample of children and adolescents with a visible difference (α = 0.81; 27).

Health-related quality of life. The EuroQol-5D-5L (EQ-5D-5L; 28) is used to assess health-related quality of life. The scale has five dimensions, namely: Mobility, Self-Care, Usual Activities, Pain/Discomfort and Anxiety/Depression. Each dimension can be assessed on five levels, which range from no problems to extreme problems. The EQ-5D-5L has good absolute and relative discriminatory power and good convergent validity(29).

Life Engagement. The Life Engagement Scale(30) will be used to measure the extent that worries or feeling bad about the way you look stop you from engaging in life activities such as going to a social event or doing sports. The scale consists of ten questions on which the adolescent can rate how much their worries or feeling bad about the way they look stopped them from doing activities on a 4-point scale. A high score indicates less life engagement. This instrument was purposively constructed for adolescents with a visible difference. The scale has good internal consistency, with $\alpha = 0.93$ for girls and $\alpha = 0.96$ for boys(30).

Appearance-related distress. The Mirror, Mirror.. (Spiegeltje, spiegeltje..; 31) questionnaire will be used to assess appearance-related distress. This questionnaire consists of 58 statements about appearance (e.g. my appearance makes me insecure), with a 5-point Likert scale ranging from "never" to "always". The questionnaire also contains a section with 38 body-parts where adolescents have to indicate on a 5-point Likert scale how happy they are with said body part (e.g. skin colour, mouth). A high score indicates more dissatisfaction with appearance. As this questionnaire is new and unpublished, it has not been validated. We aim to validate this questionnaire using the data obtained in this study.

For peer review only

230 *Depressive symptoms.* The Child Depression Inventory – 2 (CDI-2; 32) will be used to
231 assess depressive symptoms. The questionnaire consists of 28 items with three answer options
232 (e.g., I am sad sometimes, I am often sad, I am always sad). The scale consists of the
233 subscales emotional problems, divided into negative mood/physical symptoms and negative
234 self-esteem, and functional problems divided into ineffectiveness and interpersonal problems.
235 A high score indicates more depressive symptoms. The questionnaire has demonstrated
236 excellent internal reliability both for 13-16 year olds (α = 0.89), and for 17-21 year olds (α =
237 0.85; 32). The scale has good test-retest reliability (r = 0.60; 32).

238 **Recruitment**

239 Participants are recruited through several channels. The first is through patient
240 organisations. Patient organisations will be approached and asked if they are willing to
241 advertise the Dutch YP Face IT study on their website and in their newsletters. If an
242 adolescent is willing to participate, he/she is encouraged to send an e-mail to the research
243 team, providing his/her contact details. The adolescent will then receive an information
244 package containing a patient information letter, an informed consent form and a response
245 card. The second channel is through our website (www.faceitvoorjongeren.nl). On our
246 website, adolescents can read information about YP Face IT and contact the research team. If
247 they do so, they will receive an information package. The third channel is through the
248 Erasmus MC – Sophia Children's hospital. Subjects will be drawn from the department of
249 Child and Adolescent Psychiatry/Psychology, the department of Plastic and Reconstructive
250 Surgery, the department of Oral and Maxillofacial Surgery and Special Dental Care, and the
251 department of Dermatology. Subjects will be informed about the study by their treating
252 doctor/clinician. It will be explicitly stated that participation is voluntary and that they can
253 withdraw from the study at any time should they wish to do so. After informing the potential
254 participant, they will receive an information package. If a potential participant does not have

For peer review only

regular appointments at the Erasmus MC – Sophia children's hospital, we will send an information package to their home address.

A response card will be included in the information package. On this response card, adolescents can indicate whether they give permission to be contacted by the research team. If the response card has not been returned within two weeks, the research team will contact the adolescent.

If participants are willing to participate, they are asked to fill in a consent form. For participants younger than 16 years, both parents are required to sign the informed consent form. After receiving the informed consent form, the researchers will include the participant in the trial and send out the first set of online questionnaires.

Participants will receive a gift card worth €10,- after completing the entire study. If a participant decides to withdraw from the study prematurely, the adolescent will not receive a gift card.

Study design

A flow chart of the RCT is depicted in Figure 1. Prior to baseline, the in- and exclusion criteria as mentioned before (with the exception of appearance-related distress) are queried in a telephone call with the parent or adolescent. Appearance-related distress is measured based on the questionnaires completed at T1 (baseline). Adolescents will receive an e-mail with a link to all questionnaires, with the exception of the CBSA. They will receive this questionnaire by post. This procedure is the same for T2 (13 weeks, direct follow-up) and T3 (25 weeks, three months follow-up).

After completing the questionnaires, screening for appearance-related distress will take place by the research team. The scores on the SAS-A, BESAA and CDI-2 are considered.

For peer review only

For the SAS-A and the BESAA adolescents that score 0.5-2.0 standard deviations above average will be included in the study. For the CDI-2 we will include adolescents scoring in the 70th to 90th percentile, as the manual states that this corresponds to subclinical symptoms of depression(32). Adolescents that score within the defined range for one or more of the questionnaires will be randomised into the study. Adolescents that score below this range will be excluded from participation. Adolescents that score above this range will be contacted by the research team to assess whether there are clinical psychological symptoms. A phone call is made to both the adolescent and one of his/her parents. During this phone call, the DSM criteria for depressive disorder, social anxiety disorder or another suspected diagnosis will be asked. If suspicions of clinical psychological symptoms arise, the adolescents is referred to the appropriate psychological care. If no clinical symptoms are present, the adolescent is randomised into the study.

Adolescents with subclinical symptoms will be randomised to either Care As Usual (CAU) or YP Face IT. Adolescents in the CAU group will receive CAU and will complete T2 at 13 weeks and T2 at 25 weeks. The CAU group will have the opportunity to complete YP Face IT after their participation in the study. Adolescents in the YP Face IT group will receive CAU and YP Face IT, and will complete questionnaires immediately after completing YP Face IT (T2; 13 weeks) and three months after completing YP Face IT (T3; 25 weeks).

Randomisation and blinding

Participants will be randomised to either the CAU or the YP Face IT group in a 1:1 ratio. Randomisation will be stratified by age (12-13, 14-15 or 16-17 years old). Randomisation will be done using a list with a random sequence, generated using a computer program. With the stratification, six different types of blocks can be formed. Each block will independently vary between four and eight randomisation pairs. The adolescents are informed

For peer review only

of the group they are randomised to through e-mail. The researcher analysing the data will be blind to the randomisation and will not be involved in monitoring the adolescents in the intervention. This researcher also supervises data collection by students, who are not blinded. The participants, the clinical psychologist and students monitoring the adolescents in the intervention will not be blind to the treatment conditions. When contacting the research team, the adolescents are explicitly instructed not to talk about the group they were allocated to.

If, through monitoring, the research team suspects that a participant experiences clinically significant symptoms, unblinding may take place. The participant will be excluded from the study and will be transferred to more appropriate care.

Participants that drop out after randomisation will be asked if they are willing to complete the questionnaires at T2 and T3. They are in no way obliged to do so.

Sample size

For the sample size calculation we applied a mixed model ANOVA procedure, with a power of 0.80, a two-sided alpha of α=0.025 (Bonferroni corrected for two primary outcome measures), a correlation between the repeated measurements of r=0.70 and three repeated linearly decreasing measurements. Bessell and colleagues(17) reported effect sizes of d = 0.83 for appearance and d = 0.80 for anxiety in a study on Face IT. However, as we are not certain that we will obtain such a large effect, we will consider a medium effect of d = 0.50. To detect a medium effect, 56 participants are needed in both groups. As drop-out is typically high in eHealth interventions (see 33, 34), we anticipated a drop-out of 50%. Therefore, 224 participants are needed in total.

Statistical analysis

For peer review only

The data will be analysed using multilevel linear regression analyses. There will be two levels in the models. The participants constitute the upper level, their repeated measures the lower level. For each outcome variable a model will be postulated using treatment group, time and interaction with treatment group as fixed effects. Time will be entered in two ways: categorical and continuous. First, for categorical we will postulate a model with T2 (direct follow-up) and T3 (three month follow-up) using T1(baseline) as reference group. Second, for continuous we will apply the logarithm of time, as generally treatments have the largest effect at start and the level of functioning stabilizes on the long run. The deviance statistic using restricted maximum likelihood will be applied to determine the covariance structure. The deviance test will be used to determine whether the more parsimonious (log) model is a too rigorous oversimplification (35). Effect sizes will be calculated from dividing differences between the primary time-point (i.e. three months) estimations and baseline by the estimated baseline standard deviation. Analyses are done on an intention-to-treat base.

Participants who do not experience subclinical symptoms at T1, and are thus not randomised, will not be included in the analyses.

Monitoring

As the risks associated with this study are minimal, monitoring will take place once a year. An independent investigator will randomly check study documents, participant selection and participant safety. As minimal risks are involved, no interim analyses will be done.

Auditing may be done by the Erasmus MC – Sophia Children's hospital.

Patient and public involvement (PPI)

The original YP Face it intervention was developed in collaboration with adolescents and the charity Changing Faces(16). For the Dutch translation, 6 adolescents and their parents have read and reviewed the translations. Prior to this RCT an acceptability and feasibility study was conducted. In this study 14 adolescents completed YP Face IT and participated in

For peer review only

an interview. Feedback was asked on the intervention, as well as on the general study procedures (i.e. information packages, questionnaires and contact with the research team). Patient associations will be invited to help develop our dissemination strategy.

Trial status

The trial described in this paper has started in September 2019. Recruitment of participants will be until November 2020. Data collection will be until May 2021, after which the data will be analysed.

ETHICS AND DISSEMINATION

Ethics, consent and permission

This study will be conducted in accordance to the Declaration of Helsinki. Research ethics approval was obtained from the Medical Ethics review Committee (MERC) Rotterdam (reference number MEC-2018-052/NL63955.078.18). Any amendments to the study protocol and (serious) adverse events will be reported to this committee.

Data management and confidentiality

To ensure anonymous processing of the data, every participant will receive a study number. All research data will be processed using only this study number. The handling of data will comply with the European General Data Protection Regulation (GDPR). The study master file and all research data will be saved for 15 years, unless participants or their parents/guardians do not give consent for saving their data.

All data will be stored in locked cabinets, or password-protected documents. The data, including the final dataset, can be accessed only by members of the research team.

Dissemination of research findings

For peer review only

Results of this study will be presented on national and international conferences and in peer-reviewed scientific journals, within the scope of the target groups. The results will also be relayed to patients through patient organisations. Participants are also given the option to sign up for a brief study report after the results are finalised. If deemed effective, YP Face IT will be implemented in hospitals in The Netherlands.

DISCUSSION

The Dutch YP Face IT study aims to test the Dutch version of YP Face IT. With this RCT we aim to retrieve data on the effectiveness of the intervention.

Strengths and limitations

A particular strength of this study is that it follows a stage model of behavioural therapies research(36) and has excellent methodological quality. Previous research has lacked methodological rigor, due to small sample sizes, not including a control group or not blinding personnel or participants(15). Due to the nature of this study, blinding of participants is not possible. However, this study does include an adequate sample size, includes a control group and blinding of relevant personnel. With this methodological quality, we hope to be able to provide evidence-base for YP Face IT and thus provide one of the first evidence-based treatments for adolescents with a visible difference(15). Furthermore, by recruiting our participants through several channels, we will be able to obtain a sample that is representative for Dutch adolescents in general rather than solely Rotterdam, where the research team is located. Because the intervention is available online, participants throughout the entire country are able to participate without adding extra burden to participating.

In addition to strengths, this study also has some limitations. First, the intervention is available for a range of conditions causing a visible difference. The overall group results might not be generalisable to one specific group. Second, participants are not blind to the

For peer review only

treatment condition. Participants are informed about the randomisation groups. They are also informed that, if randomised to the CAU group, they will have the possibility to access YP Face IT after the study. This is a more ethical option than not offering treatment after the study and this knowledge may help keep attrition low. However, waiting list designs may lead to an overestimate of the treatment effect(37). Furthermore, this study includes only self-report measures. Diagnostic interviews or parent-reports could provide more enriched data. However, due to time and financial constraints these data will not be collected.

For peer review only

DECLARATIONS

Author statement. JMEO, HW and EMWJU developed the study design. RT made the statistical analysis plan for the study. JMEO initiated the study and obtained funding. SGMAP, EWCA, IMJM, MJK, RT, MHJH and EMWJU reviewed and supported the funding application. JMEO and EMWJU supervise the study and data collection. MvD conducts the data collection, along with psychology and medical students. MvD wrote the first draft of the manuscript. All authors read and approved the final manuscript.

Funding. This work was supported by a grant from the Innovatiefonds Zorgverzekeraars [grant number B 17-133/Dossier 3446]. The funding body has no role in the design of the study and the collection, analysis and interpretation of the data during this study.

Availability of data and material. Data sharing is not applicable to this article as no new data were created or analysed in this manuscript.

Competing interests. None declared.

Acknowledgements. Not applicable.

For peer review only

Reference list

1. Changing Faces. Prevalence of disfigurement 2010 [Available from: http://admin.changingfaces.org.uk/downloads/FE%20Campaign,%20Epidemiology%202%20pages.pdf.

2. Berger ZE, Dalton LJ. Coping with a cleft: psychosocial adjustment of adolescents with a cleft lip and palate and their parents. The Cleft Palate-Craniofacial Journal. 2009;46(4):435-43. doi:10.1597/08-093.1

3. Díaz-Atienza F, Gurpegui M. Environmental stress but not subjective distress in children or adolescents with alopecia areata. Journal of Psychosomatic Research. 2011;71(2):102-7. doi:10.1016/j.jpsychores.2011.01.007

4. Feragen KB, Kvalem IL, Rumsey N, Borge AIH. Adolescents with and without a facial difference: The role of friendships and social acceptance in perceptions of appearance and emotional resilience. Body Image. 2010;7(4):271-9. doi:10.1016/j.bodyim.2010.05.002

5. Thompson A, Kent G. Adjusting to disfigurement: processes involved in dealing with being visibly different. Clinical Psychology review. 2001;21(5):663-82. doi:10.1016/S0272-7358(00)00056-8

6. Rumsey N, Harcourt D. Visible difference amongst children and adolescents: issues and interventions. Developmental Neurorehabilitation. 2007;10(2):113-23. doi:10.1080/13638490701217396

7. Barankin B, DeKoven J. Psychosocial effect of common skin diseases. Canadian Family Physician. 2002;48:712-6.

8. Magin PJ, Pond CD, Smith WT, Watson AB, Goode SM. A cross-sectional study of psychological morbidity in patients with acne, psoriasis and atopic dermatitis in specialist dermatology and general practices. Journal of the European Academy of Dermatology and Venereology. 2008;22(12):1435-44. doi:10.1111/j.1468-3083.2008.02890.x

For peer review only

441 9. Pope AW, Snyder HT. Psychosocial Adjustment in Children and Adolescents With a
442 Craniofacial Anomaly: Age and Sex Patterns. The Cleft Palate-Craniofacial Journal.
443 2005;42(4):349-54. doi:10.1597/04-043r.1
444 10. Bakker A, Maertens KJP, Van Son MJM, Van Loey NEE. Psychological
445 consequences of pediatric burns from a child and family perspective: A review of the
446 empirical literature. Clinical Psychology Review. 2013;33(3):361-71.
447 doi:10.1016/j.cpr.2012.12.006
448 11. Turner SR, Rumsey N, Sandy JR. Psychological aspects of cleft lip and palate.
449 European Journal of Orthodontics. 1998;20(4):407-15. doi:10.1093/ejo/20.4.407
450 12. Bessell A, Moss TP. Evaluating the effectiveness of psychosocial interventions for
451 individuals with visible differences: a systematic review of the empirical literature. Body
452 Image. 2007;4(3):227-38. doi:10.1016/j.bodyim.2007.04.005
453 13. Muftin Z, Thompson AR. A systematic review of self-help for disfigurement:
454 effectiveness, usability, and acceptability. Body Image. 2013;10(4):442-50.
455 doi:10.1016/j.bodyim.2013.07.005
456 14. Norman A, Moss TP. Psychosocial interventions for adults with visible differences: a
457 systematic review. PeerJ. 2015;3:e870. doi:10.7717/peerj.870
458 15. Jenkinson E, Williamson H, Byron-Daniel J, Moss TP. Systematic Review:
459 Psychosocial Interventions for Children and Young People With Visible Differences
460 Resulting From Appearance Altering Conditions, Injury, or Treatment Effects. Journal of
461 Pediatric Psychology. 2015;40(10):1017-33. doi:10.1093/jpepsy/jsv048
462 16. Williamson H, Griffiths C, Harcourt D. Developing young person's Face IT: Online
463 psychosocial support for adolescents struggling with conditions or injuries affecting their
464 appearance. Health Psychology Open. 2015;2(2):1-12. doi:10.1177/2055102915619092

For peer review only

17. Bessell A, Brough V, Clarke A, Harcourt D, Moss TP, Rumsey N. Evaluation of the effectiveness of Face IT, a computer-based psychosocial intervention for disfigurement-related distress. Psychology, Health & Medicine. 2012;17(5):565-77. doi:10.1080/13548506.2011.647701

18. La Greca AM, Lopez N. Social anxiety among adolescents: Linkages with peer relations and friendships. Journal of Abnormal Child Psychology. 1998;26(2):83-94. doi:10.1023/A:1022684520514

19. Inderbitzen-Nolan HM, Walters KS. Social anxiety scale for adolescents: Normative data and further evidence of construct validity. Journal of Clinical Child Psychology. 2000;29(3):360-71. doi:10.1207/S15374424JCCP2903_7

20. Mendelson BK, Mendelson MJ, White DR. Body-esteem scale for adolescents and adults. Journal of Personality Assessment. 2001;76(1):90-106. doi:10.1207/S15327752JPA7601_6

21. Cragun D, DeBate RD, Ata RN, Thompson JK. Psychometric properties of the Body Esteem Scale for Adolescents and Adults in an early adolescent sample. Eating and Weight Disorders. 2013;18(3):275-82. doi:10.1007/s40519-013-0031-1

22. Haubrock I. Body-self-unity: Gender difference concerning the body self unity. Do the genders differ in the evaluation of their self and body? [Bachelor thesis]. Enschede: University of Twente; 2009.

23. Harter S. Self-perception profile for adolescents: Manual and questionnaires. In: Denver Uo, editor. Denver2012.

24. Treffers PDA, Goedhart AW, Veerman JW, Van den Bergh BRH, Ackaert L, De Rycke L. Competentie belevingsschaal voor Adolescenten. Tijdschrift voor Psychiatrie. 2004;7:468-9.

For peer review only

25. Lawrence JW, Fauerbach JA, Heinberg LJ, Doctor M, Thombs BD. The reliability and validity of the Perceived Stigmatization Questionnaire (PSQ) and the Social Comfort Questionnaire (SCQ) among an adult burn survivor sample. Psychological Assessment. 2006;18(1):106-11. doi:10.1037/1040-3590.18.1.106

26. Lawrence JW, Rosenberg L, Rimmer RB, Thombs BD, Fauerbach JA. Perceived stigmatization and social comfort: validating the constructs and their measurement among pediatric burn survivors. Rehabilitation Psychology. 2010;55(4):360-71. doi:10.1037/a0021674

27. Masnari O, Schiestl C, Rossler J, Gutlein SK, Neuhaus K, Weibel L, et al. Stigmatization predicts psychological adjustment and quality of life in children and adolescents with a facial difference. Journal of Pediatric Psychology. 2013;38(2):162-72. doi:10.1093/jpepsy/jss106

28. Herdman M, Gudex C, Lloyd A, Janssen MF, Kind P, Parkin D, et al. Development and preliminary testing of the new five-level version of EQ-5D (EQ-5D-5L). Quality of Life Research. 2011;20(10):1727-36. doi:10.1007/s11136-011-9903-x

29. Janssen MF, Pickard AS, Golicki D, Gudex C, Niewada M, Scalone L, et al. Measurement properties of the EQ-5D-5L compared to the EQ-5D-3L across eight patient groups: a multi-country study. Quality of Life Research. 2013;22(7):1717-27. doi:10.1007/s11136-012-0322-4

30. Diedrichs PC, Atkinson MJ, Steer RJ, Garbett KM, Rumsey N, Halliwell E. Effectiveness of a brief school-based body image intervention ‘Dove Confident Me: Single Session’when delivered by teachers and researchers: Results from a cluster randomised controlled trial. Behaviour Research and Therapy. 2015;74:94-104. doi:10.1016/j.brat.2015.09.004

For peer review only

31. Okkerse JME, Dessens AB. Spiegeltje, spiegeltje.. Vragenlijst voor adolescenten en volwassenen over (on)tevredenheid met het lichaam en gerelateerde lijdensdruk. 2016.

32. Kovacs M. CDI-2. Screeningsvragenlijst voor depressie bij kinderen en jongeren. Nederlandse bewerking door Denise Bodden CBeYS, editor. Amsterdam: Hogrefe Uitgevers BV; 2016.

33. Eysenbach G. The Law of Attrition. J Med Internet Res. 2005;7(1):e11. doi:10.2196/jmir.7.1.e11

34. Meyerowitz-Katz G, Ravi S, Arnolda L, Feng X, Maberly G, Astell-Burt T. Rates of Attrition and Dropout in App-Based Interventions for Chronic Disease: Systematic Review and Meta-Analysis. J Med Internet Res. 2020;22(9):e20283. doi:10.2196/20283

35. Singer JD, Willett JB. Applied longitudinal data analysis: Modeling change and event occurrence: Oxford university press; 2003.

36. Rounsaville BJ, Carroll KM, Onken LS. A Stage Model of Behavioral Therapies Research: Getting Started and Moving on From Stage I. Clinical Psychology: Science and Practice. 2001;8(2):133-42. doi:10.1093/clipsy.8.2.133

37. Cunningham JA, Kypri K, McCambridge J. Exploratory randomized controlled trial evaluating the impact of a waiting list control design. BMC Medical Research Methodology. 2013;13(1):150. doi:10.1186/1471-2288-13-150

For peer review only

Figure 1. Flow chart of the study design

For peer review only

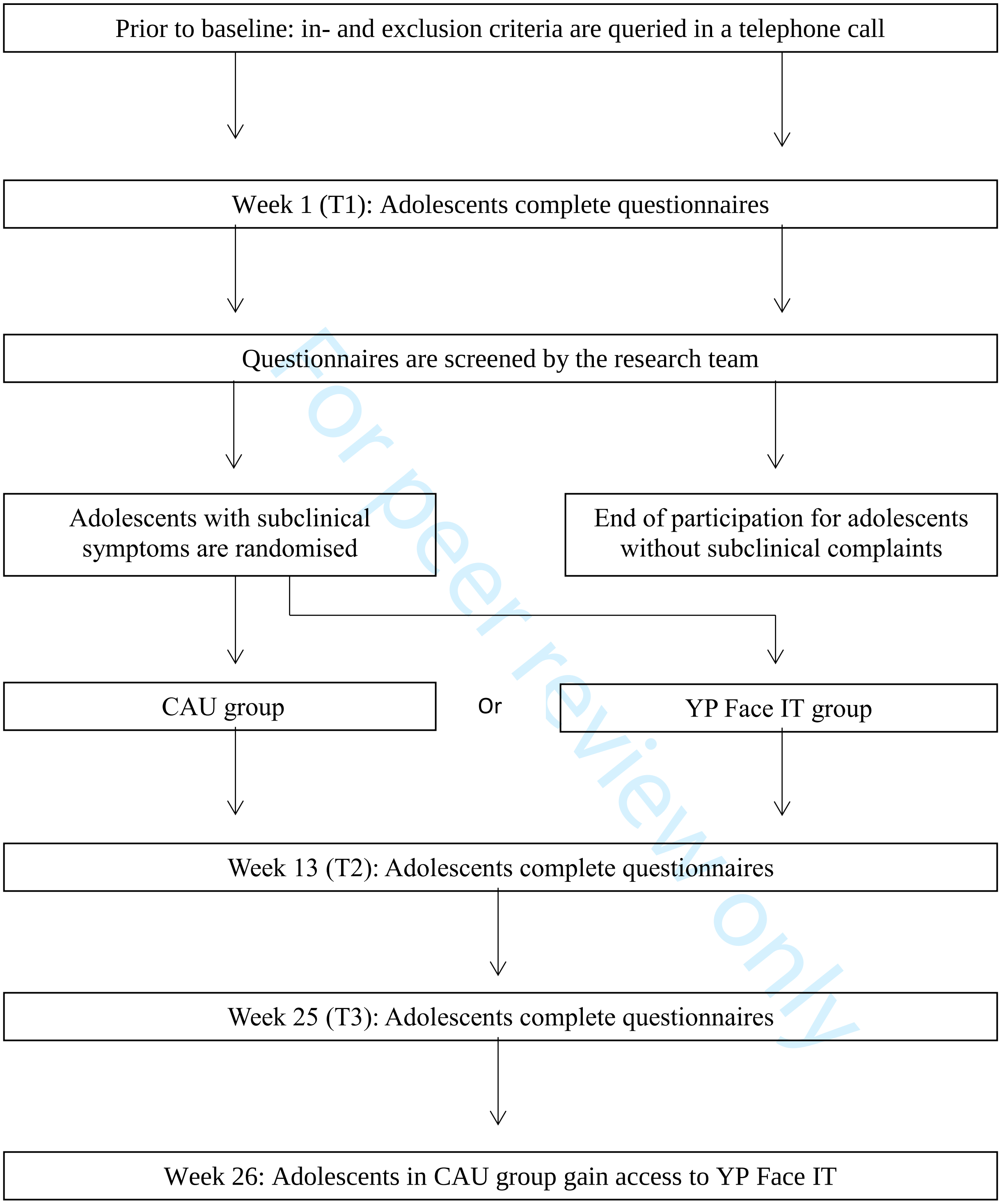

Figure 1. Flow chart of the study design

For peer review only

STANDARD PROTOCOL ITEMS: RECOMMENDATIONS FOR INTERVENTIONAL TRIALS

SPIRIT 2013 Checklist: Recommended items to address in a clinical trial protocol and related documents*

Section/item	Item No	Description	Addressed on page number
**Administrative information**			
Title	1	Descriptive title identifying the study design, population, interventions, and, if applicable, trial acronym	1
Trial registration	2a	Trial identifier and registry name. If not yet registered, name of intended registry	3
	2b	All items from the World Health Organization Trial Registration Data Set	In trial registry
Protocol version	3	Date and version identifier	page header
Funding	4	Sources and types of financial, material, and other support	20
Roles and responsibilities	5a	Names, affiliations, and roles of protocol contributors	1, 20
	5b	Name and contact information for the trial sponsor	20
	5c	Role of study sponsor and funders, if any, in study design; collection, management, analysis, and interpretation of data; writing of the report; and the decision to submit the report for publication, including whether they will have ultimate authority over any of these activities	20
	5d	Composition, roles, and responsibilities of the coordinating centre, steering committee, endpoint adjudication committee, data management team, and other individuals or groups overseeing the trial, if applicable (see Item 21a for data monitoring committee)	n/a

For peer review only

Introduction

Background and rationale	6a	Description of research question and justification for undertaking the trial, including summary of relevant studies (published and unpublished) examining benefits and harms for each intervention	4,5,6
	6b	Explanation for choice of comparators	7,9
Objectives	7	Specific objectives or hypotheses	5,6
Trial design	8	Description of trial design including type of trial (eg, parallel group, crossover, factorial, single group), allocation ratio, and framework (eg, superiority, equivalence, noninferiority, exploratory)	13-15

Methods: Participants, interventions, and outcomes

Study setting	9	Description of study settings (eg, community clinic, academic hospital) and list of countries where data will be collected. Reference to where list of study sites can be obtained	6,12-13
Eligibility criteria	10	Inclusion and exclusion criteria for participants. If applicable, eligibility criteria for study centres and individuals who will perform the interventions (eg, surgeons, psychotherapists)	6
Interventions	11a	Interventions for each group with sufficient detail to allow replication, including how and when they will be administered	6-9
	11b	Criteria for discontinuing or modifying allocated interventions for a given trial participant (eg, drug dose change in response to harms, participant request, or improving/worsening disease)	15
	11c	Strategies to improve adherence to intervention protocols, and any procedures for monitoring adherence (eg, drug tablet return, laboratory tests)	7
	11d	Relevant concomitant care and interventions that are permitted or prohibited during the trial	6
Outcomes	12	Primary, secondary, and other outcomes, including the specific measurement variable (eg, systolic blood pressure), analysis metric (eg, change from baseline, final value, time to event), method of aggregation (eg, median, proportion), and time point for each outcome. Explanation of the clinical relevance of chosen efficacy and harm outcomes is strongly recommended	9-12
Participant timeline	13	Time schedule of enrolment, interventions (including any run-ins and washouts), assessments, and visits for participants. A schematic diagram is highly recommended (see Figure)	13-14/Figure 1

For peer review only - http://bmjopen.bmj.com/site/about/guidelines.xhtml

Sample size	14	Estimated number of participants needed to achieve study objectives and how it was determined, including clinical and statistical assumptions supporting any sample size calculations	15
Recruitment	15	Strategies for achieving adequate participant enrolment to reach target sample size	12-13

Methods: Assignment of interventions (for controlled trials)

Allocation:

Sequence generation	16a	Method of generating the allocation sequence (eg, computer-generated random numbers), and list of any factors for stratification. To reduce predictability of a random sequence, details of any planned restriction (eg, blocking) should be provided in a separate document that is unavailable to those who enrol participants or assign interventions	14,15
Allocation concealment mechanism	16b	Mechanism of implementing the allocation sequence (eg, central telephone; sequentially numbered, opaque, sealed envelopes), describing any steps to conceal the sequence until interventions are assigned	14,15
Implementation	16c	Who will generate the allocation sequence, who will enrol participants, and who will assign participants to interventions	14,15
Blinding (masking)	17a	Who will be blinded after assignment to interventions (eg, trial participants, care providers, outcome assessors, data analysts), and how	14,15
	17b	If blinded, circumstances under which unblinding is permissible, and procedure for revealing a participant's allocated intervention during the trial	14,15

Methods: Data collection, management, and analysis

Data collection methods	18a	Plans for assessment and collection of outcome, baseline, and other trial data, including any related processes to promote data quality (eg, duplicate measurements, training of assessors) and a description of study instruments (eg, questionnaires, laboratory tests) along with their reliability and validity, if known. Reference to where data collection forms can be found, if not in the protocol	10-12
	18b	Plans to promote participant retention and complete follow-up, including list of any outcome data to be collected for participants who discontinue or deviate from intervention protocols	15

Data management	19	Plans for data entry, coding, security, and storage, including any related processes to promote data quality (eg, double data entry; range checks for data values). Reference to where details of data management procedures can be found, if not in the protocol	17
Statistical methods	20a	Statistical methods for analysing primary and secondary outcomes. Reference to where other details of the statistical analysis plan can be found, if not in the protocol	16
	20b	Methods for any additional analyses (eg, subgroup and adjusted analyses)	16
	20c	Definition of analysis population relating to protocol non-adherence (eg, as randomised analysis), and any statistical methods to handle missing data (eg, multiple imputation)	16
**Methods: Monitoring**			
Data monitoring	21a	Composition of data monitoring committee (DMC); summary of its role and reporting structure; statement of whether it is independent from the sponsor and competing interests; and reference to where further details about its charter can be found, if not in the protocol. Alternatively, an explanation of why a DMC is not needed	16
	21b	Description of any interim analyses and stopping guidelines, including who will have access to these interim results and make the final decision to terminate the trial	16
Harms	22	Plans for collecting, assessing, reporting, and managing solicited and spontaneously reported adverse events and other unintended effects of trial interventions or trial conduct	17
Auditing	23	Frequency and procedures for auditing trial conduct, if any, and whether the process will be independent from investigators and the sponsor	16
**Ethics and dissemination**			
Research ethics approval	24	Plans for seeking research ethics committee/institutional review board (REC/IRB) approval	17
Protocol amendments	25	Plans for communicating important protocol modifications (eg, changes to eligibility criteria, outcomes, analyses) to relevant parties (eg, investigators, REC/IRBs, trial participants, trial registries, journals, regulators)	17

Consent or assent	26a	Who will obtain informed consent or assent from potential trial participants or authorised surrogates, and how (see Item 32)	12-13
	26b	Additional consent provisions for collection and use of participant data and biological specimens in ancillary studies, if applicable	n/a
Confidentiality	27	How personal information about potential and enrolled participants will be collected, shared, and maintained in order to protect confidentiality before, during, and after the trial	17
Declaration of interests	28	Financial and other competing interests for principal investigators for the overall trial and each study site	20
Access to data	29	Statement of who will have access to the final trial dataset, and disclosure of contractual agreements that limit such access for investigators	17
Ancillary and post-trial care	30	Provisions, if any, for ancillary and post-trial care, and for compensation to those who suffer harm from trial participation	n/a
Dissemination policy	31a	Plans for investigators and sponsor to communicate trial results to participants, healthcare professionals, the public, and other relevant groups (eg, via publication, reporting in results databases, or other data sharing arrangements), including any publication restrictions	17-18
	31b	Authorship eligibility guidelines and any intended use of professional writers	n/a
	31c	Plans, if any, for granting public access to the full protocol, participant-level dataset, and statistical code	n/a
**Appendices**			
Informed consent materials	32	Model consent form and other related documentation given to participants and authorised surrogates	In appendix
Biological specimens	33	Plans for collection, laboratory evaluation, and storage of biological specimens for genetic or molecular analysis in the current trial and for future use in ancillary studies, if applicable	n/a

*It is strongly recommended that this checklist be read in conjunction with the SPIRIT 2013 Explanation & Elaboration for important clarification on the items. Amendments to the protocol should be tracked and dated. The SPIRIT checklist is copyrighted by the SPIRIT Group under the Creative Commons "Attribution-NonCommercial-NoDerivs 3.0 Unported" license.

For peer review only - http://bmjopen.bmj.com/site/about/guidelines.xhtml
